# A Survey of Flow Cytometry Data Analysis Methods

**DOI:** 10.1155/2009/584603

**Published:** 2009-12-06

**Authors:** Ali Bashashati, Ryan R. Brinkman

**Affiliations:** Terry Fox Laboratory, British Columbia Cancer Agency, Vancouver, BC, Canada V5Z 1L3

## Abstract

Flow cytometry (FCM) is widely used in health research and in treatment for a variety of tasks, such as in the diagnosis and monitoring of leukemia and lymphoma patients, providing the counts of helper-T lymphocytes needed to monitor the course and treatment of HIV infection, the evaluation of peripheral blood hematopoietic stem cell grafts, and many other diseases. In practice, FCM data analysis is performed manually, a process that requires an inordinate amount of time and is error-prone, nonreproducible, nonstandardized, and not open for re-evaluation, making it the most limiting aspect of this technology. This paper reviews state-of-the-art FCM data analysis approaches using a framework introduced to report each of the components in a data analysis pipeline. Current challenges and possible future directions in developing fully automated FCM data analysis tools are also outlined.

## 1. Introduction

Flow cytometry (FCM) is widely used in health research and treatment for a variety of tasks, such as providing the counts of helper-T lymphocytes needed to monitor the course and treatment of HIV infection, in the diagnosis and monitoring of leukemia and lymphoma patients, the evaluation of peripheral blood hematopoietic stem cell grafts, and many other diseases [1–8]. The technology is also used in cross-matching organs for transplantation, research involving stem cells, vaccine development, apoptosis, phagocytosis, and a wide range of cellular properties including phenotype, cytokine expression, and cell-cycle status [9–14]. Clinically, FCM is also used to analyze a wide array of immunological parameters in disease and to study the humoral and cellular response to vaccines. 

FCM traditionally has been a tube-based technique limited to small-scale laboratory and clinical studies [15]. Due to recent hardware advances it is now possible to analyze thousands of samples per day. This has dramatically increased the efficiency and use of this technique and allowed the adoption of FCM to high-throughput settings. 

It is widely recognized that data analysis is by far one of the most challenging and time-consuming aspects of FCM experiments as well as being a primary source of variation in clinical tests [7, 9, 10, 16–25]. Investigators have traditionally relied on intuition rather than on standardized statistical inference in the analysis of FCM data. The increased volume and complexity of FCM data resulting from the increased throughput greatly boosts the demand for reliable statistical methods and accompanying software implementations, for the analysis of these data [1–6, 16, 20, 23, 26–31]. This is because the ability to analyze FCM data is lagging far behind the ability to collect samples and to run FCM analyses, to the detriment of health research. 

This article reviews published approaches for FCM data analysis in the context of a framework created to facilitate the reporting and review process.

## 2. Background

### 2.1. FCM Data Analysis

In FCM, intact cells and their constituent components are tagged with fluorescently conjugated monoclonal antibodies and/or stained with fluorescent reagents and then analyzed individually by a flow cytometer. In the instrument, hydrodynamic forces align the cells and the fluorescent molecules in/on each cell are excited by passing through the laser light at speeds exceeding 70 000 cells per second. Each cell passing through the beam also scatters light providing an indication of cell shape and size. A flow cytometer is capable of measuring up to 20 cell characteristics, for up to millions of individual cells per sample aliquot [26, 32]. This technology can be used to examine many cellular parameters on live or fixed cells, including surface, cytoplasmic, and nuclear proteins, DNA, RNA, reactive-oxygen species, intracellular pH, and calcium flux. Measurement of the expression of cellular-activation markers, intracellular cytokines, immunological signaling, and cytoplasmic and nuclear cell cycle and transcription factors can also be readily performed [9, 11, 12, 27, 28, 33–35]. 

Typical FCM data analysis involves

1. gating (i.e., identification of homogenous cell populations that share a particular function),
2. interpretation (i.e., finding (or using) correlations between some characteristics of the identified cell populations (e.g., percentages of cells in a cell population, median fluorescent intensity of a cell population for different markers) and clinical outcomes (e.g., diagnosis, survival).

**Figure 1:**
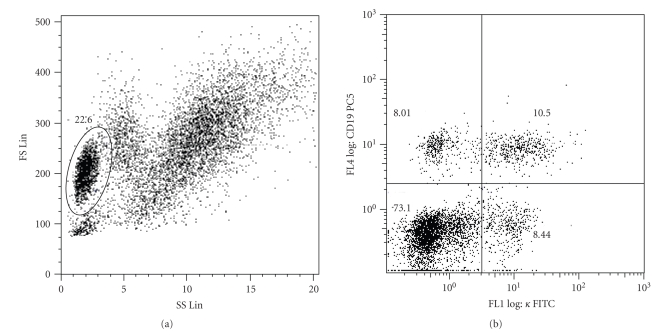
Two-dimensional sequential gating example. (a) Operator selects a subset of “interesting” events (shown within the ellipsoid region), (b) Selected events in (a) are observed and further analyzed using other dimensions of the data. The axes represent different parameters representing physical and chemical characteristics of the analyzed cells.

Gating is a highly subjective process in which the investigators determine the regions in multiparametric space that contain the “interesting” data, based on their knowledge of the experimental factors and experience (Figure 1(a)). This is a tedious, time-consuming, and often inaccurate task typically accomplished using proprietary software provided by instrument manufacturers to serially select regions in one- and two-dimensional graphical representations of the data. Intersections or unions of polygonal regions in hyperspace are then used to filter data and define a subset or subpopulation of events for further analysis (Figure 1(b)). This low-dimensional subsetting ignores the high-dimensional multivariate nature of the data. While a variety of technical issues can confound the accurate positioning of gates, even relatively minor differences in gating can produce different quantitative results [36]. A recent study involving 15 institutions shows that the mean interlaboratory coefficient of variation ranged from 17–44%, even though the same samples and reagents were used and the preparation of samples was standardized. Even though all analyses were conducted by individuals with expertise in flow cytometry, most of the variation was attributed to gating [36]. 

### 2.2. Supervised and Unsupervised Learning Techniques

Supervised and unsupervised learning techniques can be used to address the problems faced in gating and interpretation of FCM experiments. 

In supervised learning, the variables under investigation can be split into two groups: explanatory variables (e.g., measurements of events in FCM data) and one or more dependent variables (e.g., cell type). The goal here is to predict the labels of the input patterns (e.g., labels of the events in FCM data). This goal can be achieved by discovering an association between the explanatory variables and the dependent variable as is done in regression analysis. Once this association is discovered through the training stage, the algorithm can predict the dependent variable for any event of unknown label. To apply supervised data mining techniques the values of the dependent variable must be known for a sufficiently large part of the data set. 

Unsupervised learning is closer to the exploratory spirit of data mining. In unsupervised learning situations all variables are treated in the same way; there is no dependent variable. However, there is still a goal to achieve. In automated gating of FCM data, the goal is to identify the events that are in the same cluster. Clusters contain groups of events that are more similar to each other than the events from other clusters. 

The dividing line between supervised learning and unsupervised learning is the same that distinguishes discriminant analysis from cluster analysis. Supervised learning requires that the target variable is well defined and that a sufficient number of its values are given. For unsupervised learning typically the target variable is either unknown or has only been recorded for too small a number of cases.

## 3. Methods of Survey

FCM data analysis designs selected for this review include papers that met the following criteria.

1. The keyword “flow cytometry” and one or more of the keywords “automated analysis”, “automated gating”, and “automated clustering” appeared in its title, abstract, or body using Google Scholar search engine.
2. The work described one or more automated/semi-automated data analysis components. Papers that presented tutorials were not included. Papers that used manual gating procedures were included only if they employed automated analysis algorithms to analyze gating results. Papers that included simple statistical tests such as Student *t*-test on manual gating results and the papers that solely applied static gates to FCM data (without any other data processing component) were also not included.
3. Only papers published in English in refereed international journals prior to March 2009 were included.

We use the framework presented in Section 3.1 to report components involved in FCM data analysis.

### 3.1. FCM Data Analysis Framework

**Figure 2:**
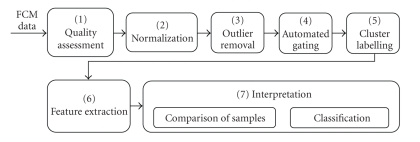
Proposed FCM data analysis framework.

Figure 2 depicts an FCM data analysis framework in which an FCM data file is analyzed through a series of analysis components. This framework has evolved from the study of FCM literature covered in this article and work in related fields, including statistics and computer science. This framework is constructed to report details of FCM data analysis studies in a systematic way to facilitate reporting and review process. The framework does not incorporate the hardware and software components used for FCM data collection.

**(1) Quality Assessment**

Artifacts from sample preparation, handling, variations in instrument parameters, or other factors may confound experimental measurements and lead to erroneous conclusions. Therefore, quality assessment is a crucial step in the use of high-throughput flow cytometry and its associated information services [37–39]. The aim of data quality assessment could include detecting whether intersample variability measurements of samples are not likely to be biologically motivated. Such samples should be identified, investigated, and potentially removed from any further analyses.

**(2) Normalization**

Like all other high-throughput data sources, there is a substantial need for normalization steps to remove nonbiological variations so that the analysis can focus on the important and relevant biological variations between samples. Instrument variability (e.g., changes in laser power), experimental protocol changes (e.g., changes in voltage setting of the instrument), and reagent changes (e.g., using antibodies from different vendors) are examples of nonbiological factors that can introduce variability in the data and shift the location of cell populations. Such changes may affect the analysis of FCM data as the main prerequisite for automated FCM data analysis is a uniform, quantitative, and comparable raw data which can be addressed by developing normalization methodologies.

**(3) Outlier Removal**

Outliers refer to observations (events in the FCM data) that deviate to such a large extent from others so as to arouse suspicion that they do not belong to the same group of observations of interest. Cell debris, dead cells, and doublets (multiple events at the same time) often contaminate FCM data and give rise to outliers. Statistics derived from data sets that include outliers may be misleading. Therefore, it is crucial to identify outliers and account for their prevalence so as to minimize their effect on subsequent analysis.

**(4) Automated Gating**

Automated identification of homogenous cell populations that share a particular function is referred to as automated gating. The main purpose of automated gating is having an objective and systematic approach for classifying cells. Automated gating can be used to

- identify known cell populations,
- discover new subpopulations of cells that might not be easily detected via standard manual gating methods. For example, cell populations may be missed due to limitations of two-dimensional manual gating.

**(5) Cluster Labelling**

Comparison of FCM samples is only possible if the same cell populations of different samples are compared against each other. For example, lymphocyte cells of two different samples can be compared against each other but it does not make sense to compare lymphocytes from one sample to granulocytes of another sample. Cluster labelling is referred to the procedure of finding similar cell populations *between* samples after automated gating. Depending on the automated gating approach used, cluster labelling may not be needed as it can be embedded in automated gating procedure. Note that similar cells *within* each sample are identified through automated gating.

**(6) Feature Extraction**

This step involves computing measurable heuristic properties (also referred to as features) of the identified gates for further analysis. Percentages of cells with respect to the total number of cells, median, and standard deviations of fluorescent intensities of different markers for the events within each gate (or gates of interest) are examples of features that can be computed for the next step.

**(7) Interpretation**

Interpretation of gating results is highly dependent on what the objective of the study is. Usually, there are two major objectives in an FCM-based study: (a) statistical comparison of samples, where the samples are compared to see if they share similar characteristics; (b) classification, where the samples are labeled to predefined classes such as healthy versus patient or patients with short survival versus the ones with long survival time. Depending on the objectives of a study (comparison versus classification), unsupervised or supervised learning techniques can be used.

## 4. Results

In Table 1, we report the data analysis components of each paper according to the framework presented in Section 3. For the papers that reported multiple designs, multiple classifications were recorded. The designs were categorized based only on what was implemented and reported in each paper. Each column in Table 1 reports the details of each of the components of the FCM data analysis framework, including the following details of each automated gating algorithm

- capability of supporting multidimensional gating,
- capability of the algorithm to determine the number of cell populations (gates) automatically,
- whether or not the algorithm belongs to the category of supervised or unsupervised learning techniques.

All the studies covered in this review (except [40, 41]) use percentages of cells within the identified gates and/or median fluorescent intensities of cell populations as the properties (features) of the identified gates for further analysis. Furthermore, a few studies address quality assessment [42–44] and normalization [44] of FCM data. Therefore, for effective use of space, Table 1 does not report the quality assessment, normalization and feature extraction components of the framework for each study. 

The entries that contain “E” refer to the term “embedded” meaning that either the cluster labelling, determining the number of cell populations, or outlier detection is embedded in the automated gating algorithm. Studies that did not implement a specific data processing component or do not have a specific capability (e.g., handling multidimensional data) have a “—” entry.

## 5. Discussion

Although a consensus among researchers for the need of a framework to describe FCM data analysis is not well documented, we feel that it can be a useful tool to facilitate research in this field. A common framework provides a reference, not only for researcher-to-researcher interaction but also for communication to persons in related fields and professions. It will also facilitate technology cross-fertilization, that is, the ability to recognize and integrate significant technological advancements made by others into one's own work. Therefore, during the course of reviewing FCM data analysis literature, we created a framework to report FCM data analysis approaches in a structured way, which facilitates the reporting and review process in the future. Our approach was to create an intuitive framework for organizing and documenting the key data analysis components described in a study and also provide a means to identify the data analysis components that have not been reported. Moreover, the use of this framework makes it easier to understand the differences between different data analysis pipelines. 

Table 1 provides a summary of the survey, making it a quick reference to review the results. For example, a quick look at the first row in Table 1 shows the design components used by Jeffries et al. [45] in their analysis of FCM data. Moreover, if somebody is interested in designing or using automated gating approaches, he/she can quickly identify the studies that address automated gating of the FCM data by referring to the third column of Table 1. The proposed framework is flexible enough to encompass the range of data analysis approaches covered in this paper. However, refining or expanding it might be necessary in the future. For example, even though a feature selection component was not needed to describe current FCM data analysis studies, addition of this component might be necessary in future. Feature selection is specifically important as it can discard the uninformative and also redundant features, facilitate data visualization and data understanding, reduce the measurement and storage requirements, reduce training and utilization times, and defy the curse of dimensionality to improve prediction performance [88]. 

**Figure 3:**
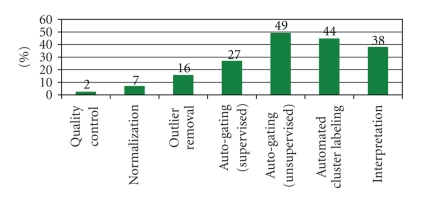
Percentages of studies that address different data analysis components according to the proposed framework. Note that cluster labeling approaches that are embedded in gating stage are counted in the “Cluster Labeling” entry.

Figure 3 shows the percentages of the studies that have addressed each of the data analysis components according to the proposed framework. 

As shown in Figure 3, most of the studies (more than 70%) focus on automated gating of FCM data from which 65% use unsupervised techniques and 35% use supervised techniques. However, only few studies focus on quality control and normalization of FCM data, suggesting that more work might still be needed in the future. 

In the rest of this section we specifically discuss the FCM data analysis methods that have been used in the context of the framework introduced in Section 3.1.

### 5.1. Quality Assessment

The basis of the quality assessment method proposed in [42, 43] is that, given a cell line, or a single sample, divided in several aliquots, the distribution of the same physical or chemical characteristics (e.g., side light scatter (SSC) or forward light scatter (FSC)) should be similar between aliquots. To test this hypothesis, five distinct visualization methods were implemented to explore the distributions and densities of ungated FCM data: Empirical Cumulative Distribution Function (ECDF) plots, histograms, boxplots, and two types of bivariate plots. Hahne et al. [44] also propose a set of visualization tools to inspect box plots of fluorescent values, number of cells, and a measure defined as “odds ratio” for similar samples within a plate. These different graphical methods provide investigators with different views of the data and can quickly flag the samples that are different from the rest. As the flagged samples may be anomalous for biological reasons, these samples are worth studying further, and some determination as to whether the sample presents data quality issues or rather presents real biological significance should be made [42].

Problems with the cell suspension, clogging of the needle, or similar issues can cause unusual patterns in the data. flowQ R package [89] addresses such problems by developing several approaches that detect disturbances in the flow of cells and also detect unusual patterns in the acquisition of fluorescence and light scatter measurements over time. These are detected dynamically by identifying trends in the signal intensity over time or local changes in the measurement intensities. The underlying hypothesis is that measurement values are acquired randomly; hence there should not be any correlation to time. Other quality assessment strategies may include investigating the number of events or the number of live cells within a sample. Furthermore, specific statistical tests addressing quality assurance requirements of an experiment can be developed. For example, in the FCM experiments to monitor clonal repopulation of engrafted single cell hematopoietic stem cells in mice [90, 91], blood samples are taken and divided into three aliquots. Each aliquot is stained with cocktail specific for detecting granulocytes/monocytes, B cells, and T cells. The percentages of each cell type from the donor population should add to roughly 100%; otherwise possible problems with the staining or the gating have occurred. Using such criterion, automated quality assurance tools can be developed to identify possible problems in the experiments.

### 5.2. Normalization

**Figure 4:**
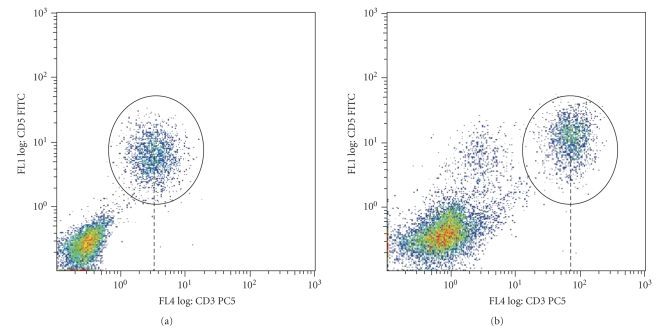
(a) and (b) Example of cases where flow cytometer voltage changes have caused in a shift in the absolute position of the populations within the ellipsoid gates.

The only study that touches on the normalization issue of FCM data proposes a method of normalizing all channels, using a model based on the size (FSC channel) of the events [44]. The authors show in their experiment that the increase in autofluorescence associated with cell size needed to be adjusted for and developed a specific linear model for this adjustment. Nonbiological variations can cause a shift or rotation in absolute position of cell populations. Figure 4 shows an example in which the voltage of the flow cytometer has changed in the channel that measures CD3 expression between the two experiments causing the population marked within the ellipsoid gate to move substantially (more than 10-fold change in median fluorescent intensity). Such variations should be accounted for during data analysis as they can cause misinterpretation of the results. For example, an ellipsoidal gate defined based on the data shown in Figure 4(a) would not capture the population of interest shown in Figure 4(b) even though the two populations represent the same cell types. While significant further developments to normalize FCM data are needed, care should be taken, as biologically motivated variations should be conserved while removing nonbiological variations.

### 5.3. Outlier Removal

Outliers can have a significant effect on automated gating results. For example, in unsupervised techniques, they can lead to overestimating the number of cell populations (i.e., clusters present in the data) needed to provide a good representation of the data. Moreover, data contaminated with outliers, when used as example data to train a supervised technique, can affect decision boundaries of the algorithm leading to poor gating results. 

Outliers can be handled in a number of ways depending on the learning technique being used. For example, in the model-based clustering framework [92, 93], they can be handled by either replacing the Gaussian distribution with a more robust one (e.g., *t* [94]) or adding an extra component to model the outliers (e.g., uniform [92]). Lo et al. [46] used a *t*-distribution in the context of model-based clustering to deal with outliers in FCM data. Jeffries et al. [45] represent two-dimensional FCM data as an image and apply a set of morphological operators on the corresponding image to remove outliers. Although Jeffries' study concentrates on two-dimensional data, the operators are applicable to multidimensional data as well. Cluster membership weights calculated during automated gating may also be used for outlier identification [30, 46]. When using supervised learning techniques, suspected examples can be removed from the learning phase to improve the generalization performance of the learning algorithm [95]. Furthermore, assigning decision confidence together with the labels of each event can be utilized to exclude the events that are less likely to belong to a specific class (e.g., [96–98]).

### 5.4. Automated Gating

More than 70% of the studies covered in this review have implemented approaches for automated gating of the FCM data. In the following subsections, we focus on these approaches in more detail. Although the approaches covered in these sections are implemented for automated gating purposes, most of them are applicable to interpretation stage of data analysis as well. 

#### 5.4.1. Supervised Techniques for Gating

Supervised techniques require training data and a training phase to learn the relationship between the events and output classes but unsupervised ones do not need this. Selection of training data that is representative of all cell populations of interest is important in training supervised techniques. Supervised techniques usually classify the input events to one of the predefined cell populations introduced to the algorithm in the training stage. Therefore, if a novel cell population exists in the data, the algorithm classifies that population as belonging to one of the predefined cell populations and not as a novel population. Two strategies can overcome this problem to some extent.

- The first one is assigning an “unknown” class for the input patterns that are unlikely to belong to known event categories [79, 96, 98]. A disadvantage of this solution is that if two novel categories exist in the test data, both will be classified as unknown even though the unknown class is comprised of multiple novel classes. It is, however, possible to add another stage of processing to further investigate the unknown events to see if they consist of multiple populations. Another similar solution would be to assume that each event can belong to several classes with different membership (e.g., event one belonging to “Class 1” with 70% chance and to “Class 2” with 30% chance) or to assign decision confidence for each classified event and reject less confident classifications as outliers or unknowns [96–98]. Using such a strategy, Wilkins et al. [75] show that more than 70% of novel species were successfully identified as “unknown” while the proportion of correctly classified species decreased moderately (from 93.8% to 86.8%) compared to the case when no novel species were identified.
- The second approach used by Beckman et al. [79] suggests adding fictitious events that reside in some of the empty spaces. Input events that are close to these fictitious events are classified as unknown events rather than being classified as belonging to the populations of interest [79]. This approach, however, needs extensive intervention in the data space in order to generate populations that represent unwanted event types. Moreover, this task is impractical when the dimension of the data is high, as one needs to generate fictitious data points that represent different unknown categories throughout the whole data space [99].

Overall, supervised techniques are suitable for tasks where we know how many classes exist in the data and a choice of unknown class would exclude the events that do not belong to the classes of interest. On the other hand, unsupervised techniques are more suitable for novel class discovery tasks. 

In supervised learning techniques, the training set should be a good representative of the future unseen data. Therefore, reproducible FCM data is necessary. For example, if there is excessive drift in the centroids of the cell populations, many of the cells could be misclassified. Some minor amount of drift can be usually accommodated by the algorithm itself and also having training sets composed of samples measured at different times for different individuals [40]. One approach to overcome this problem is to normalize the data before gating. 

Care should be taken when using supervised techniques, as usually unequal numbers of training patterns of each class are available, and this can bias the training of the classifier towards the classes with higher number of training events. One solution that has been suggested and applied to FCM data is to take into account a posteriori probabilities and class probabilities (i.e., the proportion of each of the cell categories in the training data) [86, 99, 100]. 

During training, a supervised learning algorithm reaches a state where, given sufficient and informative data, it should be capable of predicting the correct label for unseen data. However, the algorithm may adjust itself to very specific features of the training data that have little relation to unseen data. In this process referred to as overfitting, the performance on the training examples is high while the performance on unseen data becomes worse. Roughly speaking, an algorithm that is overfit is like a botanist with a photographic memory who, when presented with a new tree, concludes that it is not a tree because it has a different number of leaves from anything he/she has seen before [101]. Overfitting can be avoided by employing techniques such as regularization and early stopping [102–104]. 

Regularization involves introducing a form of penalty for complexity of the classification model. An example of regularization in neural networks is *weight decay* algorithm used in MLP neural networks. As large weights can decrease the performance of an MLP classifier on unseen data, *weight decay* penalizes the large weights causing the weights to converge to smaller absolute values than they otherwise would [102]. This strategy has been used in the context of gating FCM data [77]. 

In early stopping, the available training data is divided into two sets, that is, a *new training* set and a validation set. In each iteration of learning, the data of the *new training* set is used to train the learning algorithm and the validation set is used to evaluate its performance. The learning phase is forced to stop once the performance on the validation set does not improve or degrades. This method can be used either interactively (based on human intervention) or automatically (based on some stopping criteria usually chosen in an adhoc fashion). As mentioned in [105], early stopping is widely used as it is easy to implement and has been reported to be superior to regularization methods in many cases (e.g., [106]).

A number of algorithms in the category of supervised techniques such as multilayer perceptron (MLP) networks (e.g., [48, 54]), radial basis function (RBF) networks (e.g., [54, 75]), and support vector machines (SVM) [80] have been used in the context of cell population identification in FCM data. 

A typical MLP network consists of a set of nodes forming the input layer, one or more hidden layers, and an output layer. The MLP network has a highly connected topology since every input node is connected to all nodes in the first hidden layer, every node in the hidden layers is connected to all nodes in the next layer, and so on. The value of each node is determined by a weighted combination of input nodes, possibly including some nonlinear activation function. 

An MLP network is trained by repeated presentation of input patterns to the network. During the training process, small iterative weight changes in the structure of the network are performed until the predicted outputs are considered close enough to desired outputs. Designing an MLP classifier is not a trivial task as one needs to determine optimal parameters of the MLP structure (e.g., number of hidden layers, number of hidden layer nodes, etc.) for each specific classification task. For most problems, one hidden layer is sufficient. Using two hidden layers rarely improves the model, and it may introduce a greater risk of converging to a local minima. The network may not be able to model complex data if inadequate number of hidden layer nodes is used. On the other hand, if too many nodes are used, the training time may become excessively long, and the network may overfit the data. In general, training an MLP is relatively slow and sometimes the algorithm gets stuck in local minima and therefore the training process has to be restarted [104]. It has been shown that if an accuracy of (1 − *e*) on a test set is desirable, the number of events in the training set, *p*, should satisfy *p* ≥ *w*/*e*, where *w* is the total number of weights in the network [107]. Hence, to obtain 90% accuracy (*e* = 0.1) on test set, the desirable number of events required in training set is at least ten times the total number of weights. While having *p ≥ w/e* is definitely desirable, it is sometimes difficult in practice to build such a large database of clinical cases. An option is to use a perturbation method to generate a large number of cases by introducing small variations in actual cases [77]. The importance of having sufficiently large training sets to cover biological variation is highlighted by the increase in overall identification success of different marine microalgae in an FCM study [86]. 

An RBF neural network typically is comprised of three layers of nodes (i.e., input, hidden and output layers). The neurons in the hidden layer contain basis functions, *usually* Gaussian transfer functions whose outputs are inversely proportional to the distance from the center of the basis function. Normally the Euclidean distance is used as the distance measure, although other distance functions are also possible. An RBF network output is formed by a weighted sum of the hidden layer neuron outputs and the unity bias. 

The parameters of an RBF network which are determined in the training stage consist of the positions of the basis function centers, the radius (spread) of the basis functions in each dimension, the weights in output sum applied to the hidden layer nodes outputs as they are passed to the summation layer, the parameters of the linear part, and so forth. 

Various methods have been used to train RBF networks. One approach first uses *k*-means clustering to find cluster centers which are then used as the centers for the RBF functions. However, *k*-means clustering is a computationally intensive procedure, and it often does not generate the optimal number of centers. Another approach is to use a random subset of the training points as the centers. 

Assuming that the data is linearly separable, among the infinite number of hyperplanes that separate the data, an SVM classifier picks the one that has the smallest generalization error. Intuitively, a good choice is the hyperplane that leaves the maximum margin between the two classes, where the margin is defined as the sum of the distances of the hyperplane from the support vectors. Support vectors are the examples closest to the separating hyperplane and the aim of an SVM classifier is to orientate this hyperplane in such a way that it is as far as possible from the closest members of both classes. If the two classes are nonseparable we can still look for the hyperplane that maximizes the margin and that minimizes a quantity proportional to the number of misclassification errors. The trade-off between margin and misclassification error is controlled by a positive constant *C* (referred to as error penalty) that has to be chosen beforehand [101, 108]. 

SVMs are very universal learners. In their basic form, SVMs learn linear threshold function. Nevertheless, by a simple “plug-in” of an appropriate kernel function, they can be extended to nonlinear classifiers such as polynomial classifiers, radial basis function (RBF) networks, and three-layer sigmoid neural networks. 

Perhaps the biggest limitation of the SVM approach lies in the choice of the kernel. Once the kernel is fixed, SVM classifiers have only one user-chosen parameter (the error penalty) [101]. 

RBF networks can be trained significantly faster than MLPs. In addition to the number of hidden layers, a difference between RBF and MLP classifiers lies in the nodes of the hidden layer, which use different kernels (basis functions) to represent the data. RBF networks have the advantage of not suffering from local minima in the same way as MLPs. While for an RBF there is no restriction on decision boundaries formed, an MLP forms convex decision boundaries. Moreover, RBF's hidden layer performs a nonlinear mapping from the input space into a (usually) higher-dimensional space in which the input patterns become linearly separable [109]. Although RBF networks are quick to train, when training is finished and it is being used, it is slower than an MLP. Therefore, where speed is a factor an MLP may be more appropriate. 

SVM can be seen as a new way to train polynomial, neural network, or RBF classifiers. While most of the techniques used to train the above mentioned classifiers are based on the idea of minimizing the training error, which is usually called *empirical risk, *SVMs operate on another induction principle, called *structural risk minimization, *which minimizes an upper bound on the generalization error [108]. 

In the context of FCM data analysis, Boddy et al. [81] compares the performances of RBF networks using different basis functions. Specifically, radially symmetric and a more general arbitrarily oriented ellipsoidal basis functions were employed, with the latter proving to be significantly superior in performance. The distance between input patterns and the basis function centers are defined by a distance metric, which determines the shape of the basis function. The Euclidean distance metric produces hyperspherical (radially symmetric) basis functions around the basis functions centers. Mahalanobis distance metric, on the other hand, allows the hyperellipsoid (nonradially symmetric) to adopt any orientation that best fits the data distributions. 

Wilkins et al. [54] compare several classification algorithms such as MLP, RBF, and LVQ (learning vector quantization) to identify phytoplankton species from FCM data. The authors show that identification success was more or less similar using the above-mentioned techniques. Therefore, they suggest using the criteria mentioned earlier and characteristics of the data at hand to decide which method is the best to use. In another study on phytoplankton species, Morris et al. [80] demonstrate that an SVM classifier outperforms RBF classification. These studies focus on specific data sets and their generalization on other data sets is unknown. Therefore, picking an algorithm based on the type of data at hand and above-mentioned characteristics of learning algorithms is recommended. One approach that might be worth considering in FCM studies is the multiple classifier systems (MCSs) [110]. MCSs are based on combining the outputs of ensembles of different classifiers (supervised learning techniques). Classification accuracy improvements are possible provided that a suitable combination function is designed and that the individual classifiers make different errors. Ideally, a combination function should take advantage of the strengths of individual classifiers, avoid their weaknesses, and improve classification accuracy [110].

#### 5.4.2. Unsupervised Techniques for Gating

Algorithms for unsupervised analysis of FCM data should be

- computationally efficient as the amount of data generated for each FCM experiment is large (an FCM experiment contains measurements for up to millions of cells for up to 20 parameters),
- able to detect clusters with different shapes as clusters (cell populations) in FCM data can have different shapes ranging from spherical shapes to irregular shapes such as being highly elongated or even being curved,
- able to detect populations with different densities and percentages as FCM samples can contain a wide range of cell populations in terms of the density of cells (very sparse vs. very dense cell populations) and also percentages of cells in each population (populations of interest as low as 0.1% of total events),
- able to determine the number of cell populations as the number of cell populations present in the data is usually not known apriori,
- able to handle outliers as data can contain significant number of outliers.

The above-mentioned characteristics of FCM data make unsupervised analysis challenging as existing clustering algorithms either do not address or have limitations in addressing these requirements. 

Clustering algorithms require the number of clusters that they should identify to be specified apriori. There are several approaches for choosing the number of clusters, including resampling, cross-validation, and various information criteria [111]. Zeng et al. [53] use the peaks of density distribution of each channel of FCM data and estimate the numbers of clusters to be identified by *k*-Means algorithm. Lo et al. [46] propose to use Bayesian information criteria (BIC) in the context of a model-based clustering approach to estimate the optimal number of clusters. BIC is computationally cheap to compute once maximum likelihood estimation for the model parameters has been completed, an advantage over other approaches, especially in the context of FCM where datasets tend to be very large. While computationally cheap, BIC relies heavily on an approximation of marginal likelihoods, which might not be very accurate for some data. Alternative approaches such as the integrated completed likelihood [112] may improve the estimation of the number of clusters. Nevertheless, combined with expert knowledge, such approaches can provide guidance on choosing a reasonable starting number of clusters. 

Sometimes it is possible that even if the actual number of clusters is known, the clustering algorithm may not identify the correct clusters at the level of separation that is desired. This can happen when there is a rare cell population within the FCM data. In this case, the clustering algorithm may consider the rare population as an outlier or as part of a larger cell population and instead divide larger cell populations into smaller populations. One approach to overcome this problem might be clustering the data with higher number of clusters with the hope that the rare populations are represented by separate clusters and use some merging algorithm to combine the clusters that are similar according to a criterion. 

*k*-means clustering algorithm is one of the methods that have been used in literature. While this approach performs well when the clusters are spherical in shape, clusters in FCM data usually are not spherical. Demers et al. [82] have proposed an extension of *k*-means allowing for nonspherical clusters, but this algorithm has been shown to lead to inferior performance compared to fuzzy *k*-means clustering [50]. In fuzzy *k*-means [113], each cell can belong to several clusters with different association degrees, rather than belonging to only one cluster. Even though fuzzy *k*-means takes into consideration some form of classification uncertainty, it is a heuristic-based algorithm and lacks a formal statistical foundation. Other choices include hierarchical clustering algorithms (e.g., linkage or Pearson coefficients method). However, these algorithms are not appropriate for FCM data, since the size of the pairwise distance matrix increases in the order of *n* ^2^ with the number of cells, unless they are applied to some preliminary partition of the data [72], or they are used to cluster across samples, each of which is represented by a few statistics aggregating measurements of individual cells [87, 114]. Since the required processing time for some clustering algorithms increases significantly by the increase in the number of events and parameters of FCM data, subsampling the data might be a suitable approach to reduce the processing time. Care should be taken when performing subsampling to make sure that the properties of the original data are preserved after this process. For example, a random uniform sampling of data may not be a suitable approach as it can discard the small populations present in the data. One alternative might be using a guided sampling approach in which representative events are selected from low-density populations as well. This might be achieved by different strategies such as looking at density distributions of the data or performing a coarse clustering before subsampling procedure. 

An alternative approach for FCM data gating is to model the FCM data with mixtures of distributions. The most commonly used model-based clustering approach is based on finite Gaussian mixture models [93, 115]. However, Gaussian mixture models rely on the assumption that each component follows a Gaussian distribution, which is often not the case when modeling FCM data. A common approach is to look for transformations of the data that make the normality assumption more realistic. Lo et al. [46] proposed the use of the Box-Cox [116] transformation prior to using a model-based clustering. In addition to nonnormality, there is also the problem of outlier identification in mixture modeling. As mentioned earlier, replacing the Gaussian distribution with a more robust one (e.g., *t* [94, 115]) or adding an extra component to model the outliers (e.g., uniform [92]) is suggested to deal with outliers. The *t*-distribution is similar in shape to the Gaussian distribution with heavier tails and thus provides a robust alternative [117]. The Box-Cox transformation is a type of power transformation, which can bring skewed data back to symmetry, a property of both the Gaussian and *t*-distributions. In particular, the Box-Cox transformation is effective for data where the dispersion increases with the magnitude, a scenario not uncommon to FCM data [46]. 

One of the benefits of model-based clustering approach is that it provides mechanism for both “hard” clustering (i.e., the partitioning of the whole data into separate clusters) and fuzzy clustering (i.e., a “soft” clustering approach in which each event may be associated with more than one cluster) [46]. The latter approach is in line with the rationale that there exists uncertainty about to which cluster an event should be assigned.

### 5.5. Cluster Labelling

Cluster labelling (or cluster matching) between samples is usually performed manually. Approaches that can label the clusters based on their location such as mean or median fluorescent intensity (MFI) of known cell populations or their location relative to other clusters have been used in literature [45]. Cluster labelling approaches that take into account the shape and rotation of cell populations in addition to their locations might provide more robust results. In case of using the absolute location of cell populations for cluster labelling, data normalization prior to labelling is necessary as significant changes in the location of cell populations (as shown in Figure 4) can result in mismatching cell populations. Note that in case of using supervised techniques for automated gating, labelling is not needed as the gating algorithm determines the labels of events (e.g., whether the events are of cell type 1 or cell type 2). Therefore, this information can be used for labelling (matching) cell populations between samples as well.

### 5.6. Feature Extraction

Prior to interpretation of gating results, features representing the identified cell populations need to be defined. In literature, usually the percentages and locations of cell populations are used for interpretation purposes. However, other characteristics of cell populations such as their shapes (e.g., whether they are spherical or ellipsoidal), dispersion, orientation, and proportion of a specific cell population relative to another cell population may also be useful to achieve better interpretation results. Since the features that may carry information are not always known apriori, one option is to generate as many features as possible and then use feature selection techniques to discard the uninformative and also redundant features. 

Furthermore, approaches such as the one introduced in [41] that uses other representations of the characteristics of the FCM data (characteristics based on kernel density estimation in the case of [41]) might be interesting to investigate further. Since the final aim in some studies such as the one presented in [41] is to perform a classification task (e.g., healthy versus patient), gating FCM data may not be necessary (except to find basic cell populations such as live cells and lymphocytes) which can potentially eliminate the errors that can be introduced in the system by poor gating strategies.

### 5.7. Interpretation

Although mostly done manually, interpretation of results can utilize many methods that have been developed in computer science for finding associations between FCM samples with their labels (e.g., disease diagnosis) or identifying cluster of patients with similar FCM data. Depending on the purpose of the study, supervised or unsupervised learning techniques can be used. For example, if the aim is to classify a sample as disease or healthy, supervised learning techniques can be used. For the purpose of finding patients who have similar data, standard unsupervised learning techniques can be utilized.

## 6. Conclusions

The need for completely automated analysis of FCM data is becoming more evident with the advances in high-throughput FCM technology. To date, most research has been focused on developing approaches for automated gating of FCM data. Manual gating is recognized as labor intensive, subjective, and prone to error when processing large numbers of samples. Therefore, automated gating methods will allow for a faster and more robust data analysis pipeline. Although significant effort is still needed to develop automated gating algorithms that address challenging aspects of FCM data, we believe that the research community needs to look beyond automated gating and develop bioinformatics tools that facilitate building *completely* automated FCM data analysis pipelines. It should be noted that the development of robust, automated methods for high-throughput FCM data analysis also requires high-quality data to feed into the analysis framework. Generating this high-quality data requires well-designed experiments with the appropriate positive and negative controls. 

A rigorous quantitative assessment is important before using automated approaches in practice, as a replacement for expert manual analysis. Moreover, it is likely that one data analysis solution may not be suitable to address specific questions of a study or address the challenges of analyzing a specific FCM dataset. For example, if somebody is interested in identifying a previously known type of cell, supervised techniques might be better suited. Overall, in order to use automated data analysis approaches in biomedical research and clinical setting, we need to develop more generic solutions or design smart algorithms that can tune themselves with little intervention, as the users may not have enough knowledge of bioinformatics techniques. The availability of a wide variety of example data is crucial, as it would aid in the development, evaluation, and comparison of different automated analysis methodologies. 

The development of automated FCM data analysis approaches will greatly facilitate both basic research and clinical applications in medical/agricultural areas that depend upon this technique. Since FCM generates data sets as complex and informative as gene arrays using markers for different cell populations defined by phenotypic, activation, or cytokine expression features, optimizing FCM-based data analysis will also help develop FCM as a proteomics and diagnostic tool with widespread applications in both basic and clinical laboratories.

## Figures and Tables

**Table 1:** Summary of survey (M: manual; Y: yes; E: embedded in gating; U: unsupervised; S: supervised; “—”: not supported, not implemented, not applicable; “||”: same as above). Note that this table does not report Quality Assessment, Normalization, and Feature Extraction components.

Paper	Outlier removal	Automated gating				Labelling	Interpretation (classification/ comparison of samples)
		Method	Supervised/ Unsupervised	Multidimensional	Automated # of clusters		
[45]	Logical and cleaning morphological operators applied to the corresponding image representation of FCM data	Logical operation on image representation of FCM data followed by thickening	U	—	—	Based on location and abundance of populations	—
	||	Majority operator applied to the image representation of FCM data followed by Soble edge detection	U	—	—	||	—
	||	Zero-degree B-Spline smoother applied to the 2-dimenisonal FCM data followed by break point detection	U	—	—	||	—
	||	Gath-Geva fuzzy clustering	U	—	—	||	—

[30]	Embedded in clustering (cluster membership weights can be used to exclude outliers)	Gaussian Mixture Models	U	Y		M	—
					Y		
					(using BIC)		


[46]	Embedded in clustering (cluster membership weights can be used to exclude outliers)	t-Mixture Models	U	Y		M	—
					Y		
					(using BIC)		


[47]	Embedded in clustering (excluding events that are far from Gaussian functions centers using a predefined cutoff value)	Mahalanobis distance from centroids of multivariate Gaussian functions used for classification task	S	—	—	E	—

[48]	—	Multilayer perceptron (MLP)	S	Y	—	E	—

[49]	—	Building templates for automated gating by using a cluster-finding algorithm (Beckton Dickinson's (BD) snap-to gate algorithm)	U	—	—	E (initially set by operator)	—

[50]	—	DKLL (an extension of the *k*-means algorithm to allow for non-spherical clusters)	U	Y	—	—	—
	—	Fuzzy *k*-means based on adaptive distance	U	Y	—	—	—
	—	Fuzzy *k*-means based on maximum likelihood	U	Y	—	—	—
	—	Fuzzy *k*-means based on minimum total volume	U	Y	—	—	—
	—	Fuzzy *k*-means based on sum of all normalized determinants	U	Y	—	—	—

[51]	—	M	—	—	—	M	Complete linkage hierarchical clustering

[52]	—	—	—	—	—	—	Comparing sample to a reference sample by probability binning algorithm

[53]	—	*k*-means	U	Y	Histogram feature guided	—	—
					Partition index guided		—

[17]	—	Frequency difference gating approach (defines a gate(s) that contains statistically significant more events in the test sample than the control sample)^1^	U	Y	—	—	—

[54]	—	MLP	S	Y	—	E	—
	—	Learning vector quantization (LVQ)	S	Y	—	E	—
	—	Radial basis function (RBF)	S	Y	—	E	—
	—	Asymetric RBF	S	Y	—	E	—
	—	Classification by modeling each class with Gaussian distributions	S	Y	—	E	—
	—	*k*-nearest neighbour method	S	Y	—	E	—
	—	Kohonen's self organizing map (SOM)	U	Y	—	M	—

[55]	—	Static gates applied to data	U	—	—	E (initially set by operator)	CLASSIF1 approach [56, 57]

[36]	—	Building templates for automated gating by using a cluster-finding algorithm (BD Snap-to gate algorithm)	U	—	—	E (initially set by operator)	—

[43]^2^	—	M	—	—	—	M	Functional linear discriminant analysis

[58]	—	Building templates for automated gating by using a cluster-finding algorithm (BD's snap-to gate algorithm)	U	—	—	E (initially set by operator)	—

[59]	—	Gaussian Mixture Models	U	—	M	M	—

[60]	—	M	—	—	—	M	Average-linkage hierarchical clustering

[61]	—	M	—	—	—	M	Classification based on a semantic network of knowledge base through a hierarchical tree (if-then rule mechanism)

[62]	—	*k*-means	U	Y	—	M	—
	—	Calculating modes of density function (calculated by Kernel density estimation ) followed by nearest neighbour heuristic	U	Y	—	M	—
	—	Gaussian mixture models using Markov chain Monte Carlo (MCMC)	U	Y	—	M	—

[63]	—	Building templates for automated gating by using a cluster-finding algorithm (BD's snap-to gate algorithm)	U	—	—	E (initially set by operator)	—

[64]	—	Automated gating using BD Simulset software	—	—	—	M	Correlation tests using Spearman's method

[65]	—	Image representation of randomly selected events from a group of flow data followed by smoothing, regional maxima detection and watershed algorithm to define the gates to apply to all the data	U	—	—	—	—

[66]	—	SOM	U	—	—	M	—
	—	Cluster analysis with Winlist (Verity Software House, USA))	U	—	—	||	—

[67]	—	Static gates applied to data and self adjusting gates (details not mentioned) for lymphocytes, monocytes, and granulocytes	U	—	—	E (initially set by operator)	CLASSIF1 approach [56, 57]

[68]	—	Fcom tool (an analysis tool in Winlist (Verity Software House, USA))	—	—	—	M	Average- linkage hierarchical clustering

[69]	—	Static gates applied to data and self adjusting gates for lymphocytes, monocytes, and granulocytes	U	—	—	E (initially set by operator)	CLASSIF1 approach [56, 57]

[70]	—	M	—	—	—	M	“Professor Fidelio” (a heuristic classification system that reasons on the basis of defined diagnostic patterns [71])

[72]	—	*k*-means followed by Murphy's cluster joining algorithm based on standard deviation of the data [73]	U	—	—	M	—
	—	*k*-means followed by a cluster joining algorithm based on modified spread of the data and modified distance between two clusters [72]	U	—	—	M	—
	—	Preclustering a subset of the data by *k*-means and assigning unclustered events to the closest cluster center followed by a cluster joining algorithm based on modified spread of the data and modified distance between two cluster [72]	U	—	—	M	—

[73]	E (excluding the events that were more than a set number of standard deviations away from the centroids of the clusters)	*k*-means followed by Murphy's cluster joining algorithm based on standard deviation of the data	U	Y	—	M	—

[74]	—	MLP	S	Y	—	E	—

[75]	—	RBF	S	Y	—	E	—

[76]	—	MLP	S	Y	—	E	—
	—	SOM	U	Y	—	M	—
	E (excluding the events that were more than a set number of standard deviations away from the centroids of the cluster)	*k*-means	U	Y	—	M	—

[77]	—	No gating—mean fluorescent intensities of antibodies were used for next stage of analysis	—	—	—	—	MLP

[78]	—	RBF	S	Y	—	E	—

[40]	—	—	—	—	—	—	Histogram of one parameter of FCM data followed by MLP

[79]	—	Classification and regression trees (CARTs)	S	Y	—	E	—

[80]	—	Support vector machine (SVM)	S	Y	—	E	—
	—	RBF	S	Y	—	E	—

[81]	—	RBF using radially symmetric basis function (based on Euclidean distance)	S	Y	—	E	—
	—	RBF using more general arbitrarily oriented ellipsoidal basis functions (based on Mahalanobis distance)	S	Y	—	E	—

[82]	—	Gaussian mixture model clustering	U	Y	—	—	—

[83]	Embedded in clustering (excluding events that are far from Gaussian functions centers using a predefined cutoff value)	Mahalanobis distance from the centroids of multivariate Gaussian functions used for classification task	S	—	—	E	—

[84]	—	M	—	—	—	—	Classification based on a shrunken centroids approach [85]
							Hierarchical clustering

[41]	—	M	—	—	—	—	Kernel density estimation followed by calculating differences between patients by Kulback-Leibler divergence to form a similarity matrix and then dimensionality reduction by multidimensional scaling for 2-dimensional visualization

[86]	—	RBF	S	Y	—	E	—

[87]	—	M	—	—	—	—	Hierarchical clustering
							Principal component analysis (PCA) for dimensionality reduction and visualization to see if classes are separable by looking at the first few principle components

^1^Closely related to probability binning algorithm introduced in [52].

^2^This study utilizes quality assessment strategy introduced in [42] that is based on comparison of density, ECDF (empirical cumulative distribution function), box plots, and two types of bivariate plots of similar samples.
